# Post-COVID-19 related osteonecrosis of the jaw (PC-RONJ): an alarming morbidity in COVID-19 surviving patients

**DOI:** 10.1186/s12879-022-07518-9

**Published:** 2022-06-14

**Authors:** Haytham Al-Mahalawy, Yehia El-Mahallawy, Noha Y. Dessoky, Sally Ibrahim, Hatem Amer, Haytham Mohamed Ayad, Hagar Mahmoud El Sherif, Alshaimaa Ahmed Shabaan

**Affiliations:** 1 Oral and Maxillofacial Surgery Department, Faculty of Dentistry, Fayoum University, Fayoum, Egypt; 2 Head of the Oral and Maxillofacial Department, Faculty of Dentistry, Fayoum University, Fayoum, Egypt; 3 Oral and Maxillofacial Surgery Department, Faculty of Dentistry, Alexandria University, Champlion st, Azrite, Alexandria Egypt; 4 Oral & Maxillofacial Pathology Department, Faculty of Dentistry, Fayoum University, Fayoum, Egypt; 5 Oral & Maxillofacial Pathology Department, Faculty of Dentistry, Cairo University, Cairo, Egypt; 6 Oral & Maxillofacial Surgery at Nasser Institute Hospital for Research & Treatment, Cairo, Egypt

**Keywords:** Post-COVID, Osteonecrosis, Maxilla, Avascular necrosis

## Abstract

**Purpose:**

The recent coronavirus disease (COVID-19) pandemic mainly affects the respiratory system; however, several oral and maxillofacial post-COVID-19 complications have also been observed. This series reports the growing number of osteonecrosis cases associated with post-COVID-19 patients.

**Materials and methods:**

This is a retrospective, multi-center case series that reports cases with maxillary osteonecrosis after various periods of SARS-CoV-2 infection in the period between January and August 2021 based on the PROCESS guidelines.

**Results:**

Twelve cases were reported with post-COVID-19 manifestation of spontaneous osteonecrosis of the maxillary jaw. Five patients were hospitalized during COVID-19 management and all of the twelve cases had at least one systematic Co-morbidity, and undertake corticosteroids prescription based on the COVID-19 disease treatment protocol. The mean onset of osteonecrosis symptoms appearance was 5.5 ± 2.43 weeks calculated from the day of the negative PCR test. The management was successfully done through surgical debridement and pre and post-operative antibiotics. No anti-fungal medications were prescribed as the fungal culture and the histopathological report were negative.

**Conclusion:**

Post-COVID-related osteonecrosis of the jaw (PC-RONJ) could be now considered as one of the potential post-COVID-19 oral and maxillofacial complications that occurs unprovokedly and mainly in the maxilla.

**Supplementary Information:**

The online version contains supplementary material available at 10.1186/s12879-022-07518-9.

## Background

As per the most recent iteration from the World Health Organization, the current situation report of the Corona-Virus-Disease-19 (COVID-19) pandemic has resulted in a cumulative total of 332,617,707 confirmed cases, including 5,551,314 deaths and 3,156,986 new cases reported globally [1]^.^ Although many patients recover from COVID-19, it is important to keep in mind that there may be complications after recovery. One such complication in the maxillofacial region is Avascular Necrosis (AVN), which may lead to deteriorating outcomes and significant tissue loss [2].

COVID-19 is caused by Severe Acute Respiratory Syndrome Corona Virus-2 (SARS-CoV-2). Viral infection pathogenicity begins with the virus targeting the Angiotensin-Converting Enzyme-Two (ACE-2) receptors, which enable viral entry into cells. ACE-2 receptors are distributed throughout the body in different tissues, including type II alveolar pneumocytes in the lungs, vascular endothelial cells, smooth muscle cells, and enterocytes within the intestines, oral and nasal mucosa [2, 3].

ACE-2 downregulation and recognition of SARS-CoV-2 spike glycoproteins by Pattern Recognition Receptors (PPPs) on cell membranes leads to activation of the innate immune system and local inflammation at the viral entry point, which ultimately results in a hyperinflammatory cytokine storm [3, 4].

Additionally, viral downregulation of ACE-2 receptors leads to endothelial dysfunction, which, together with viral-induced hyperinflammation, promotes endothelial dysfunction and endotheliitis, with subsequent microvascular dysfunction not only at the viral entry points but also in several organs. This cascade of events demonstrates that SARS-CoV-2 promotes a hypercoagulable state through unique mechanisms and interactions between thrombosis and inflammation [3, 4].

It has been postulated that direct vulnerability of the oral mucosa to SARS-CoV-2 infection leads to COVID-19-associated oral manifestations. These manifestations are reported to be a consequence of the high ACE-2 expression in the epithelial cells of the oral mucosa [5, 6]. The aim of this case series is to report the growing number of osteonecrosis cases, mainly involving the maxilla, associated with Post-COVID-19 survival patients.

## Patients and methods

This study reports a retrospective, multi-center, single country, case series to demonstrate the clinical characteristics of patients burdened with maxillary osteonecrosis behavior with a history of a recent SARS-CoV-2 infection according to the PROCESS Guideline [7]. The included patients in this study were referred to oral and maxillofacial surgeon for consultation regarding a painful intraoral condition in the period between January and August 2021. All patients had previously undergone polymerase chain reaction (PCR) testing for SARS-CoV-2, which confirmed infection.

The elapsed period between the SARS-CoV-2 infection and the emergence of the oral and maxillofacial symptoms was reported by the patients, along with the manner of COVID-19 infection management, whether corticosteroids were prescribed, and the patients’ medical history.

**Fig. 1:**
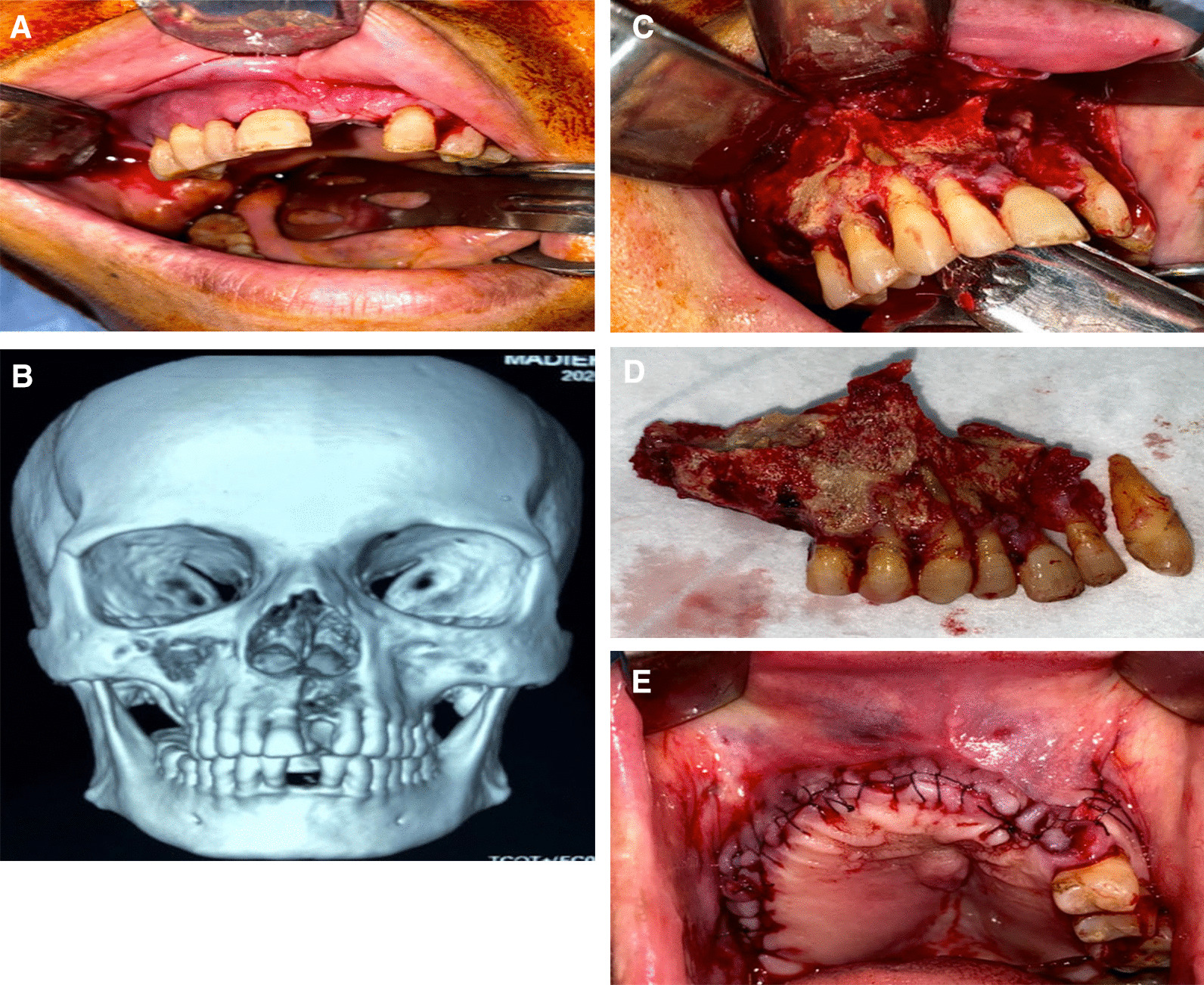
Report of a case with Post-COVID-19 Related osteonecrosis of the Jaw (PC-RONJ). **A** Photograph showing maxillary multiple buccal pus oozing fistulae. **B** Three-Dimensional volumetric rendering of a Computed Tomography (CT)-Scan showing necrotic right maxillary segment extending to the right maxillary sinus and toward the maxillary left lateral incisor. **C** Necrotic bone exposure via sulcular incision. **D** Extent of the resected segment of the maxilla. **E** Postoperative defect extent and wound water-tight suturing

The chief complaints of the patients were reported, along with a detailed clinical examination to localize the infection, and a confirmatory radiographic examination (Computed Tomography [CT]-Scan) to delineate the extent of the affection. The CT image also showed the affection of the maxillary sinus, and whether it was obliterated or clear. Any provoking stimulus in the post-COVID infection was reported, including recent dental treatment, any type of trauma, or wearing a removable partial denture (Fig. 1). A microbiological mucosal cotton swab was used to designate the causative microorganism and its sensitive antibiotic agent. The specimen was examined under microscope and then incubated for bacterial culture on blood agar, chocolate agar, and MacConkey agar (Biolab Diagnostics Laboratory Inc, Budapest, Hungary) under aerobic and anaerobic atmosphere for 48 h. A fungal culture was incubated on sabouraud dextrose agar for 7 days to label any fungal infection.

Management of the patient comorbidities was performed before the start of the surgical phase. All patients signed informed consent before the start of the treatment protocol. All of the reported subjects were hospitalized and prepared for general anesthesia, where surgical debridement was performed. The necrotic maxilla was exposed via a sulcular incision, and the margins between the vital bone and the sequestrum were detected. Separation and debridement of the dead bone were performed, followed by smoothing of any sharp bony edges, copious saline irrigation, and water-tight suturing. The specimen was sent for histopathology analysis. The patients were put under culture-specific postoperative antibiotic coverage for a minimum of 3 weeks based on the clinical pharmacist’s recommendation.

The study was based on the declaration of Helsinki recommendations. All of the investigated patients have signed an informed consent before embarking in this study after clarifying the study purpose, risks, and benefits (Additional file 1: Video S1).

## Results

This case series reports twelve cases of Post-COVID oral manifestations with both peculiar and common clinical behavior. Table 1 summarizes the report of these cases. The mean age of the cases was 56.1 ± 9.65 (37–68) years, and seven of them (58.3%) were females. While none of the cases were admitted to the Intensive Care Unit (ICU) during SARS-CoV-2 infection management, five of them were hospitalized for various periods and seven were treated through home isolation. All of the reported patients undertake corticosteroids prescription based on the COVID-19 disease treatment protocol. The five hospitalized patients were given 6 mg injection of Dexamethasone every 24 h for ten days, while the seven home isolated cases were given 6 mg oral Dexamethasone for 10 days. None of the disclosed patients reported any history of head and neck radiotherapy, bisphosphonate or other antiresorptive or antiangiogenic medications intake.

**Table 1:** Reported cases with Post-COVID related osteonecrosis of the Jaw (PCRONJ)

	Age	Sex	Comorbidities	COVID Hospitalization, ICU or home isolation	CS intake	Microbial culture/isolated organism	Time between COVID infection and onset of OM	Site	Main symptoms	Spontaneous or provoked ONJ (extraction/ ill-fitting denture)	Treatment	Post-op AB (period)	Follow-up
1	61	F	DM	Hospitalization	+ ve	Bacterial Staphylococci	8 W	Maxilla	Palatal swelling, alveolar crest pus oozing fistula, maxillary da mobility	Spontaneous	Surgical debridement	+ ve (3 W)	U/E
2	66	F	DM, hypertensive	Hospitalization	+ ve	Bacterial G-ve Bacilli	4 W	Maxilla	Palatal swelling, buccal pus oozing fistula, maxillary DA mobility	Spontaneous	Surgical debridement	+ ve (5 W)	U/E
3	37	F	DM	Home isolation	+ ve	Bacterial G-ve Bacilli	12 W	Maxilla	Exposed necrotic bone, Alveolar crest pus oozing fistula	Spontaneous	Surgical debridement	+ ve (3 W)	U/E
4	68	M	DM, hypertensive, atrial fibrillation	Hospitalization	+ ve	Bacterial Neisseria spp	4 W	Maxilla	Palatal swelling, Alveolar crest pus oozing fistula, maxillary DA mobility	Spontaneous	Surgical debridement	+ ve (4 W)	U/E
5	55	M	DM	Home isolation	+ ve	Bacterial G-ve Bacilli	4 W	Maxilla	Maxillary DA mobility	Spontaneous	Surgical debridement	+ ve (3 W)	U/E
6	59	F	DM, hypertensive	Hospitalization	+ ve	Bacterial G-ve Bacilli	6 W	Maxilla	Alveolar crest pus oozing fistula, Maxillary DA mobility	Spontaneous	Surgical debridement	+ ve (4 W)	U/E
7	63	M	DM, hypertensive	Home isolation	+ ve	Bacterial G-ve Bacilli	3 W	Maxilla	Exposed necrotic bone, alveolar crest pus oozing fistula, maxillary DA mobility	Spontaneous	Surgical debridement	+ ve (4 W)	U/E
8	49	M	DM	Home isolation	+ ve	Bacterial G-ve Bacilli	5 W	Maxilla	Maxillary DA mobility	Spontaneous	Surgical debridement	+ ve (4 W)	U/E
9	55	F	DM	Home isolation	+ ve	Bacterial G-ve Bacilli	5 W	Maxilla	Exposed necrotic bone, Alveolar crest pus oozing fistula	Spontaneous	Surgical debridement	+ ve (5 W)	U/E
10	53	M	DM	Hospitalization	+ ve	Bacterial G-ve Bacilli	4 W	Maxilla	Maxillary DA mobility	Spontaneous	Surgical debridement	+ ve (5 W)	U/E
11	65	F	DM, Hypertensive	Home Isolation	+ ve	Bacterial G-ve Bacill	5 W	Maxilla	Exposed necrotic bone	Spontaneous	Surgical debridement	+ ve (6 W)	U/E
12	42	F	DM	Home Isolation	+ ve	Bacterial Enterococcus spp	6 W	Maxilla	Maxillary DA mobility	Spontaneous	Surgical debridement	+ ve (3 W)	U/E

*ONJ* osteo necrosis of the jaw, *M* male; *F* female; *DM* diabetes mellitus; *CS* cortico-steroids; + ve, Positive; −ve, Negative; spp, several species; *W* week; *DA* Dento-Alveolar; *ICU* Intensive Care Unit; *Post-op* postoperative; *AB* antibiotic; *U/E* uneventful

All of the cases showed a mutual clinical picture and maxillary exclusivity of osteonecrosis with spontaneous, unprovoked, occurrence following various periods of COVID-19 disease and its management. The mean onset of jaw necrosis development was 5.5 ± 2.43 (3–12) weeks, calculated from the day of the negative PCR test. No precipitating event or provoking factor were reported in the period prior to the onset of the osteonecrosis symptoms in any of the twelve reported patients.

**Fig. 2:**
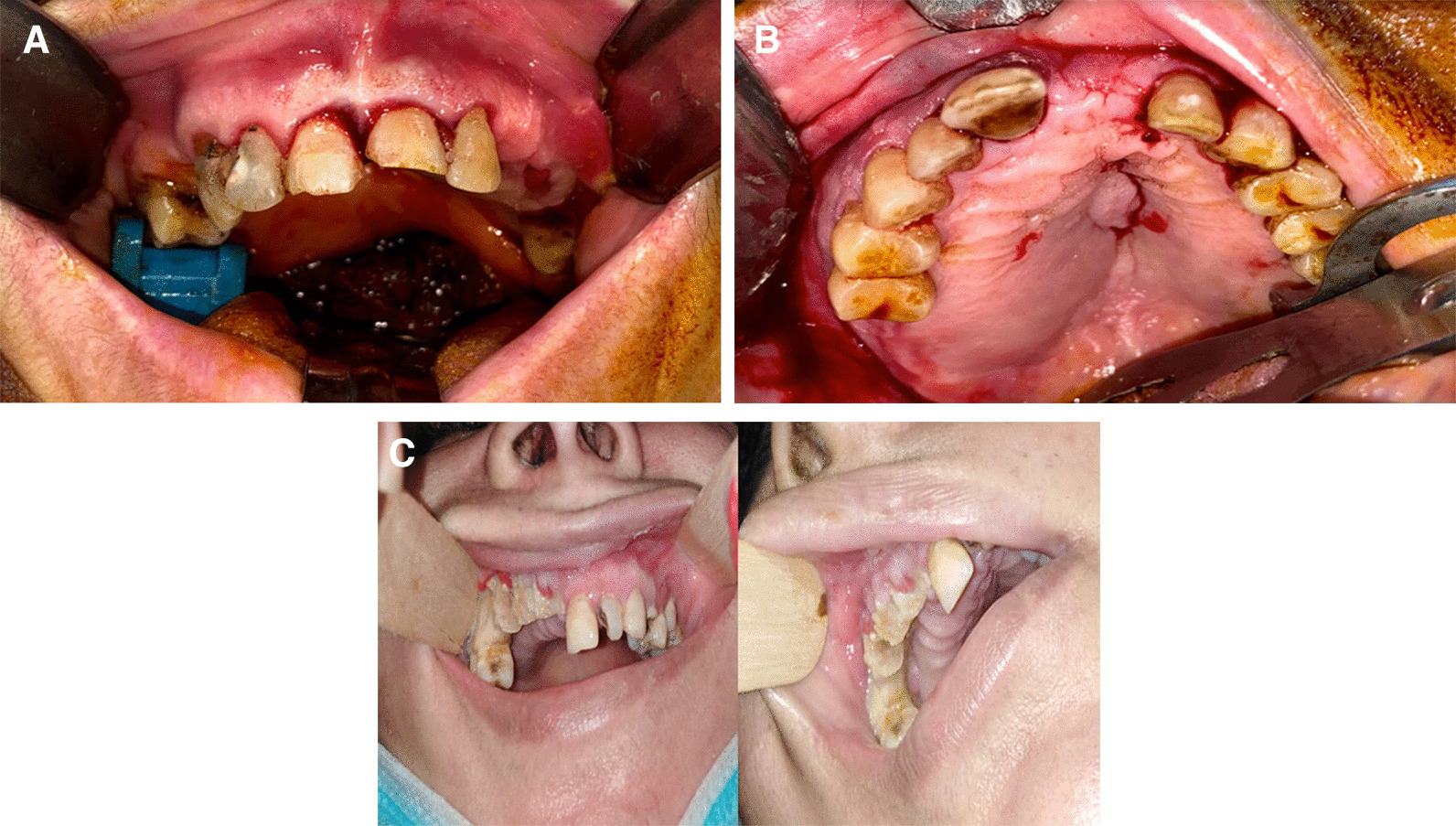
Variant clinical picture, which may be presented in patients with Post-COVID-19 Related osteonecrosis of the Jaw (PC-RONJ). **A** A mobile dentoalveolar maxillary segment with intact mucosal bone coverage. **B** Palatal swelling. **C** Mucosal ulceration and exposed necrotic bone

Regarding the clinical manifestations, a mobile dentoalveolar maxillary segment was observed in nine patients, a pus-oozing fistula in eight patients, palatal swelling in three patients, and exposed necrotic bone with mucosal ulceration in four patient (Fig. 2).

The fungal culture and microscopic examination came out to be negative in all of the reported cases, indicating the absence of any fungal infestation or growth. Bacterial growth was detected from the bacterial-culture swab of all of the reported cases. A stained smear of gram negative, ampicillin and sulbactam resistant bacilli was isolated in nine swabs. In the remaining three swabs, *Enterococcus* spp, *Neisseria* spp, and Staphylococci were isolated respectively.

A common surgical protocol was proposed to all of the study patients, where a surgical debridement was performed, followed by culture-based antibiotic therapy for a minimum period of 3 weeks. No antifungal agent was prescribed in any of the reported subjects. All of the patients were followed for a minimum of 2 months, during which, no recurrence or propagation of the inflammatory processes was reported. A confirmatory C-reactive protein (CRP) blood test revealed normal values in all cases.

**Fig. 3:**
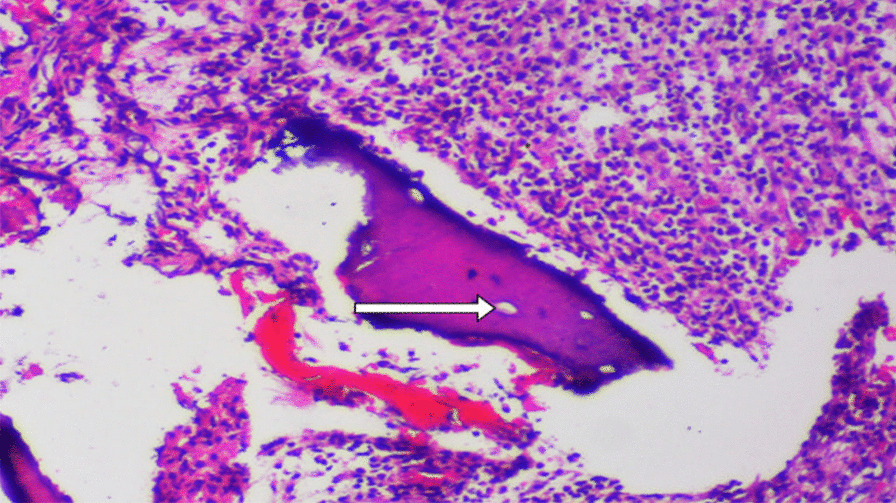
H&E Photomicrograph view (× 400) showing sequestrated bony trabeculae with empty osteocytic lacunae (white arrow) and heavy inflammatory cells infiltration, together with areas of hemorrhage and dystrophic calcification

A recurring histopathological picture was reported in all cases. Examination of Hematoxylin and Eosin-Stained (H&E) sections demonstrated bony trabeculae with numerous necrotic fragments and empty osteocytic lacunae. The bone marrow showed heavy infiltration of inflammatory cells, mainly composed of lymphocytes, histiocytes, plasma cells, and dense sheets of polymorphous and osteoclasts giant cells. This chronically inflamed fibrous tissue was found along with areas of hemorrhage and dystrophic calcification (Fig. 3). No fungal invasion of bone trabeculae was reported in any of the cases. Confirmatory Periodic acid–Schiff-Stained (PAS) sections showed no fungal infiltration.

## Discussion

**Fig. 4:**
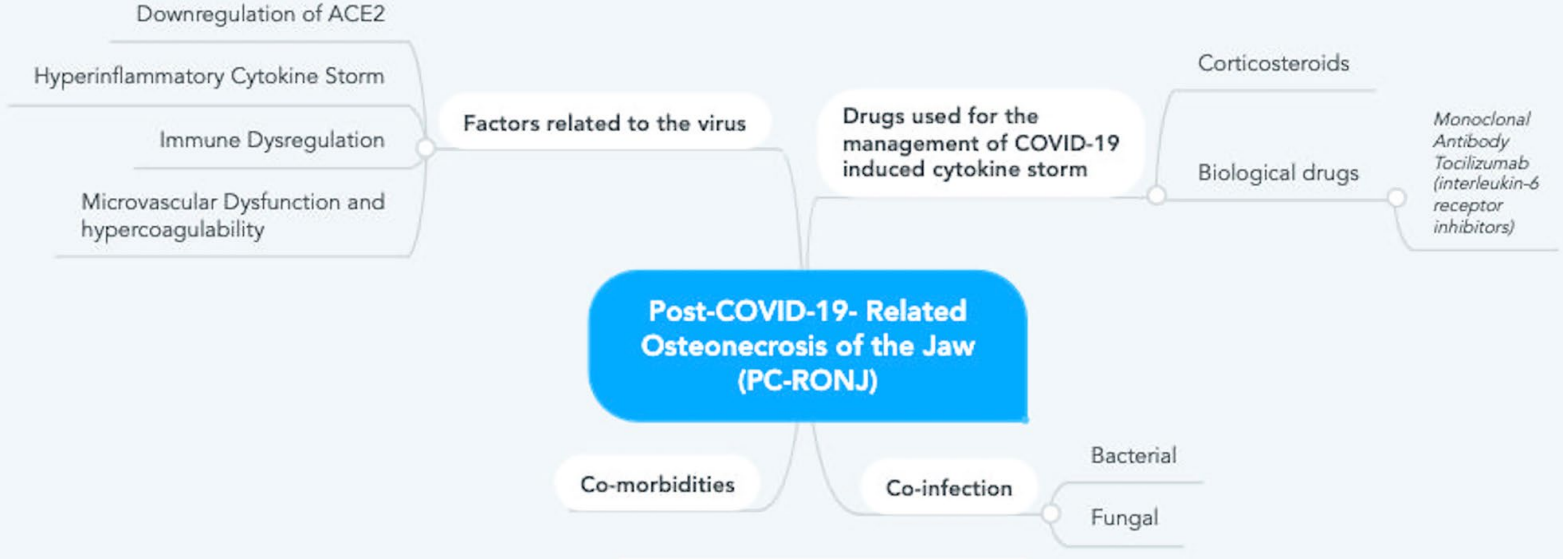
Flowchart outlining potential Post-COVID-19 Related osteonecrosis of the Jaw (PC-RONJ) predisposing factors

Many factors have the potential to contribute to the induction and propagation of osteonecrosis of the jaw related to post COVID-19 infection. The main factor is the virus itself, which causes ACE-2 downregulation and subsequent hyperinflammatory state, with elevated cytokines and immune dysregulation, in addition to microvascular thromboses and the hypercoagulability state [3, 4]. The second factor is the drugs used for the management of the hyperinflammatory syndrome and cytokine storm, which are the corticosteroids and the biological drugs like the Monoclonal Antibody Tocilizumab [8, 9]. The third factor is the co-infections, which could be either bacterial or fungal secondary infections [10–12]. The fourth factor is the associated co-morbidities, especially diabetes, which further impair the local and innate body immunity [13, 14]. All these factors are directly linked to the development of jaw osteonecrosis, either independently or as a result of their interactions. An arbitrary statement of these factors is presented in Fig. 4.

This is a report of twelve SARS-CoV-2 infection-survived cases with manifestations of maxillary jaw osteonecrosis, which developed after the infectious course of the disease over various periods. None of the patients had fungal infection, confirmed by negative preoperative fungal swab, negative histopathological reports, and the treatment consisted of surgical debridement and antibiotics without any antifungal medications. The mean time until the diagnosis of osteonecrosis was 5.5 ± 2.43 weeks after recovery from SARS-CoV-2 infection. This time frame is in accordance with Boymuradov et al., who reported a minimal elapsed period of 2 months after SARS-CoV-2 infection to develop the symptoms of osteonecrosis, and a maximum symptom-free period of 8 months [15].

All patients in this study received corticosteroids for the treatment of COVID-19. Recent studies on COVID-19 recovered patients have shown the increased risk of developing osteonecrosis with the use of glucocorticoids [16, 17]. Daltro et al. (2020) conducted a literature review to assess the relationship between COVID-19 and osteonecrosis, where the findings of eleven studies related COVID-19 to osteonecrosis and the use of corticosteroids; therefore, they concluded that there is sufficient evidence for a corticosteroid-associated risk of avascular necrosis in patients with COVID-19 [18]. The unjustified utilization of corticosteroids as a component of the COVID-19 treatment protocol is a common practice without scientific support, especially in mild to moderate cases. Moreover, Wang et al. conducted a meta-analysis to demonstrate the proportion and effect of corticosteroid therapy in patients with COVID-19 infection, where they deduced that a delayed viral clearance was a hallmark in patients with COVID-19 who received corticosteroid therapy with no clearly improved survival rate [19]. Therefore, the use of corticosteroids in the management of SARS-CoV-2 infection must be balanced between its advantage and post-infection drawbacks and complications. As a result, the use of corticosteroids should be restricted to severe cases and only during acute respiratory symptoms.

All of the included patients have additional comorbidities; where all of them were diabetic. Several reports have linked the association between COVID-19 in patients with diabetes with the occurrence of necrosis of the jaws and other bones [15, 18, 20, 21]. Microvascular angiopathy is a well stated complication of diabetes, which impairs bone nutrition through loss of full function of the nutrient vessels in bone. Increased blood sugar level has a direct effect on white blood cells chemotaxis and phagocytosis, which subsequently improves viral binding affinity and reduce virus clearance [18, 20]. Shin et al. (2020) reported that physicians should manage diabetes with proactive strategies to minimize the risk of medical complications in SARS-CoV-2 affected patients [20].

Interestingly, all of the reported cases which considered to have a potential relationship between COVID-19 and osteonecrosis of the jaw were exclusively found in the maxilla. This may be attributed to the close proximity to the nasal mucosa and maxillary sinus. Furthermore, the high expression of ACE-2 receptors in the nasal and oral mucosa epithelial cells may act as a vital risk factor for the occurrence of decreased blood supply [5, 6]. It is speculated that a sinusitis-induced osteomyelitis is the commencement point for this aggressive condition as SARS-CoV-2 is a respiratory tract infection.

Bagaria (2021), in an attempt to explain those peculiar orthopedic post-COVID manifestations, postulated that aggressive SARS-CoV-2 infection depletes immune defense mechanism, which leaves the patient vulnerable to infection episodes. This phenomenon is called ‘Consumption Immuno-compromisation’. In his report, Bagaria (2021) reported the occurrence of two cases of spontaneous osteonecrosis of the long bone and two cases of acute primary septic joint, along with a high rate of postoperative infection [22]. All of the bony manifestations that are encountered in this study developed spontaneously, without any provoking events. This spontaneous incidence of bone necrosis is a cause of concern and should be a point of additional analysis.

Several authors have reported the spontaneous occurrence of jawbone necrosis without any precipitating events or predisposing factors [23, 24]. Khominsky and Lim (2018) conducted a systematic review of the spontaneous occurrence of Medication-Related Osteonecrosis of the Jaw (MRONJ), where they concluded that the “spontaneous” nomenclature is a misnomer as most of the literature-reported cases are the sequelae of low-grade trauma to thin susceptible mucosa covering anatomical regions at-risk areas [23].

To the best of our knowledge, this is the first series of reported cases with maxillary osteonecrosis following SARS-CoV-2 infection. This article is limited by its observational pilot nature and limited sample size. The exact pathogenesis of jaw necrosis, and whether the virus contributing effect alone can initiate pathological vascular necrosis without the ramifying effect of steroid therapy or patients comorbidity are topics for future research. Furthermore; the decrease in blood supply to the affected hard and soft tissues of the maxilla could not be evaluated in this study. A prophylactic approach should be presented for the early detection of bone osteonecrosis by the assessment of the vasculature of the maxillary hard and soft tissues in suspected patients.

Finally, several recurring risk factors must be built before considering a patient to have PC-RONJ. A recent SARS-CoV-2 infection with concurrent cumulative corticosteroid prescription, systematic co-morbid conditions, no history of current or previous radiotherapy, antiresorptive, or antiangiogenic agents intake all of which are considered as precipitating factors for the occurrence of un-provoked jawbone osteonecrosis.

A practical guidelines is called for the prevention, diagnosis, and management of jaw osteonecrosis in patients with recent SARS-CoV-2 infection. With the increasing rate of COVID-19, the general dentists and oral and maxillofacial surgeons must be best prepared and positioned for the early identification and prevention of PC-RONJ to avoid permeant deformity. There is a need for communication between oral and maxillofacial surgeons and physicians to reduce the severity and the vast degree of avoidable morbidity that may occur as a consequence of an unjustified corticosteroid prescription, where they can modify the COVID-19 treatment protocol. A specialized follow-up plan for the early detection of PC-RONJ should be proposed for patients at risk of developing osteonecrosis, as the spontaneous nature of this condition occurs with silent impact.

## Conclusion

Jaw osteonecrosis occurrence in patients recovered from SARS-COV-2 infection is of a potential concern. Medically compromised patients and those who are prescribed corticosteroids are considered as significant risk factors. Future studies are required to confirm whether the virus itself or the other factors lead to jaw osteonecrosis.

## Supplementary Information

**Additional file 1.** A video document demonstrating a mobile dento-alveolar segment.

## Data Availability

All data generated or analysed during this study are included in this published article.

## Abbreviations

COVID-19: Corona-virus-disease-19
AVN: Avascular necrosis
SARS-CoV-2: Severe acute respiratory syndrome corona virus-2
ACE-2: Angiotensin-converting enzyme-two
PPPs: Pattern recognition receptors
PCR: Polymerase chain reaction
CT: Computed tomography
ICU: Intensive care unit
PAS: Periodic acid–Schiff-stained
H&E: Haematoxylin and eosin
PC-RONJ: Post-COVID-19 related osteonecrosis of the Jaw
MRONJ: Medication-related osteonecrosis of the jaw
